# Effectiveness and Safety of a Moisturizer Containing Centella Asiatica Extract, Ceramide NP, and Panthenol for Erythema and Skin Barrier in Erythematotelangiectatic Rosacea: A Randomized Controlled Trial Study

**DOI:** 10.1111/srt.70396

**Published:** 2026-10-15

**Authors:** Xia Jiang, Haoyun Luo, Xiao He, Chenmin Hu, Kena Dan, Yongchao Dang, Tian Qian, Daojun Zhang, Fei Hao

**Affiliations:** ^1^ Dermatology and Plastic Surgery Center The Third Affiliated Hospital of Chongqing Medical University Chongqing China

**Keywords:** erythema, erythematotelangiectatic rosacea, moisturizer, Rosacea‐specific Quality‐of‐life instrument, safety, skin barrier

## Abstract

**Background:**

Erythematotelangiectatic rosacea is a chronic recurrent inflammatory disease that often impose a significant psychological burden on patients. Given that effective moisturization is integral to its management, the choice of a proper moisturizer is a critical clinical consideration. Our study aims to assess the effectiveness and safety of a moisturizer containing Centella asiatica extract, ceramide NP, and panthenol for erythema and skin barrier in Chinese patients with erythematotelangiectatic rosacea.

**Methods:**

All enrolled patients were prescribed oral minocycline 50 mg once daily and applied moisturizer twice daily. Facial images of facial redness, stratum corneum hydration (SCH), transepidermal water loss (TEWL), and Clinician's Erythema Assessment (CEA) were collected at baseline, day 7 (D7), day 14 (D14), and day 28 (D28). Time to a 1‐point reduction in the Patient's Self‐Assessment (PSA) scale and Rosacea‐specific Quality‐of‐life instrument (RosQol) were also recorded by patients.

**Results:**

A total of 75 patients with rosacea were enrolled, 39 patients were randomized to the experimental group and 36 to the control group. Our study showed that the variations of a* value, facial red area, SCH and RosQol from baseline to D28 were rapidly and significantly greater in the experimental group, and the mean rank of CEA was significantly higher in the control group at D28. All AEs were mild and self‐limiting.

**Conclusions:**

This study found that the moisturizer containing Centella asiatica extract, ceramide NP, and panthenol delivers rapid, effective adjunctive benefits in rosacea treatment and represents a reliable daily skincare option for patients.

## 1 Introduction

Rosacea, a chronic recurrent inflammatory disease primarily affecting the central face, typically manifests as persistent or intermittent flushing, telangiectasia, papules and pustules, along with stinging, burning or pain. Sensitivity in the affected skin involves multiple factors, including abnormal immune responses, vascular and neurogenic dysregulation and compromised skin barrier [1, 2]. The prevalence of rosacea is approximately 3.22%–5.46% worldwide, while it is about 3.4% in China [3, 4, 5, 6, 7]. Data from studies indicate that erythematotelangiectatic rosacea (ETR) is the most common subtype of rosacea, accounting for up to 47.6%–56.7% of cases [7, 8]. Furthermore, persistent erythema has been identified as the most frequent diagnostic feature in rosacea patients, accounting for up to 78.3% of all diagnostic features, and it is also the most bothersome symptom, significantly impairing quality of life across domains of self‐perception, emotional well‐being, daily grooming, and social functioning [9, 10].

The management of persistent erythema in rosacea typically involves systemic therapies like minocycline or doxycycline, laser treatment and daily skincare [1]. Major guidelines or consensus, such as the guideline of National Rosacea Society Expert Committee of Unite States, the German S2k guideline, and the Asia‐Pacific expert consensus, highlight a core three‐step skincare regimen: cleansing, moisturization, and photoprotection (CMP) [1, 4, 11, 12]. Among these, moisturization is essential. An ideal moisturizer should be fragrance free, alcohol free, hypoallergenic, and non‐acnegenic, while also strengthening the skin barrier, boosting hydration, soothing irritation, and enhancing tolerance to external aggressors [11, 13, 14].

Cetaphil soothing and comforting cream has been specially developed for sensitive skin which meets the criteria for rosacea skincare recommended in the Asia‐Pacific expert consensus. Its key active ingredients include Centella asiatica extract (CAE), panthenol, and ceramide NP. Previous studies have reported that, CAE has moisturizing and anti‐inflammatory properties [15, 16], Panthenol could enhance stratum corneum hydration and reduce transepidermal water loss [17, 18], and Ceramide NP replenishes intercellular lipids and maintains epidermal integrity [19]. Data from the studies by Su et al. [20] and Dimmer et al. [21] showed that Cetaphil soothing and comforting cream could significantly reduce skin sensitivity, alleviate facial redness, and repair the skin barrier in individuals with sensitive skin. In our study, patients with ETR were recruited and treated with a combination of minocycline and moisturizers for 28 days, and we aimed to evaluate the additional benefit of Cetaphil soothing and comforting cream in this patient population.

## 2 Materials and Methods

This study was a randomized controlled trial study which was registered in the Chinese Clinical Trial Registry and conducted in accordance with the Declaration of Helsinki. Written informed consent was obtained from all patients before the start of the study. The research procedure flowchart is presented in Figure 1.

**FIGURE 1:**
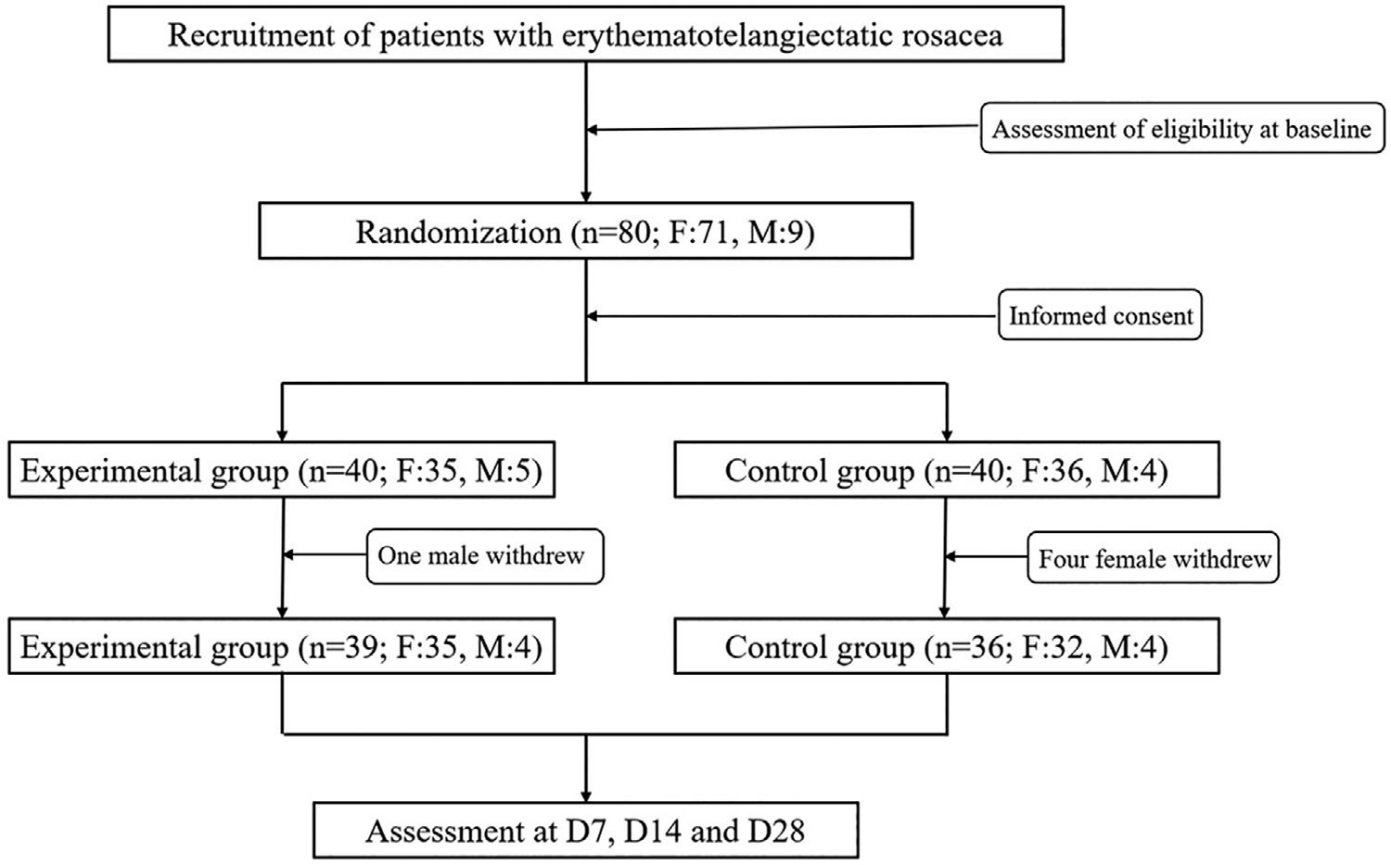
The research procedure flowchart.

### 2.1 Study Population

All patients were aged 18–50 years, had a dermatologist‐confirmed diagnosis of erythematotelangiectatic rosacea (ETR) with persistent erythema (Clinician's Erythema Assessment score 2–4), and were considered suitable for minocycline treatment. Patients were excluded if they were pregnant or lactating, had an allergy to the study moisturizer, had a history of severe systemic disease (including oncologic, coagulation, hepatic, or cardiovascular/cerebrovascular disorders) or any neuropsychiatric disorder that could preclude compliance, or had used topical medications within 2 weeks, had used systemic medications or received aesthetic treatments within 1 month, or had participated in any other clinical trial within 3 months prior to enrollment.

### 2.2 Patient Randomization and Treatment Procedures

A total of 80 patients with ETR were enrolled in this study, comprising 71 females and 9 males. All enrolled patients were randomized 1:1 to the experimental or control groups using simple randomization, based on a randomization list generated by an independent statistician using SAS 9.4 software (SAS Institute Inc., Cary, NC, USA). During the study, 4 females and 1 male withdrew due to adverse events (AEs). Consequently, 75 patients completed the study, with 39 in the treatment group (35 females and 4 males) and 36 in the control group (32 females and 4 males). The experimental group received the Cetaphil soothing and comforting cream, while the control group received the Cetaphil daily hydrating lotion which served as a basic moisturizer. All patients were prescribed oral minocycline 50 mg once daily and applied the assigned moisturizer twice daily, in addition, patients were instructed to cleanse their face daily with Cetaphil Soothing Foam Wash and to adhere strictly to physical sun protection measures.

### 2.3 Materials and Clinical Assessment

At baseline and on day 7 (D7), day 14 (D14), and day 28 (D28), the following data were collected: Facial photographs (front, left 45°, and right 45°) of the patients routinely captured using VISIA‐CR 5.0(Canfield Scientific Inc., Fairfield, New Jersey) [22], stratum corneum hydration (SCH) measured on the cheek using a Corneometer CM 825(Courage + Khazaka electronic GmbH, Köln, Germany) [23], transepidermal water loss (TEWL) measured on the cheek using a Tewameter TM Hex(Courage + Khazaka electronic GmbH, Köln, Germany) [24], and Clinician's Erythema Assessment (CEA, 0 = clear, 1 = almost clear, 2 = mild, 3 = moderate, and 4 = severe) evaluated by a dermatologist [25]. Additionally, the time to a 1‐point reduction in the Patient's Self‐Assessment (PSA) scale and the scores of on the Rosacea‐specific Quality‐of‐life instrument (RosQol) were also recorded by patients [26, 27].

Facial redness was evaluated based on the a* value and the facial red area derived from facial photographs, and these parameters were analyzed using Image‐Pro Plus 6.0 (Media Cybernetics, Inc., Silver Spring, USA) and VISIA‐CR 5.0(Canfield Scientific Inc., Fairfield, New Jersey) respectively [22].

### 2.4 Safety Evaluation

Patients were instructed to contact the study dermatologist via WeChat at any time if they experienced any facial discomfort (e.g., pain, itching, prickling, erythema, papules, or vesicles) while using the cream.

### 2.5 Statistic Analysis

We analyzed sample characteristics by using medians and interquartile ranges for continuous variables and frequencies and percentages for categorical variables. Bivariate associations were assessed using the non‐parametric Mann–Whitney U test or Kruskal–Wallis test for continuous variables and the chi‐square or Fisher's exact tests for categorical variables. Correlations between CEA and time points were studied using the Spearman's rank correlation (correlation degree: 0–0.19, very weak; 0.20–0.39, weak; 0.40–0.59, moderate; 0.60–0.79, strong; 0.80–1.00, very strong). Differences in CEA between the experimental and control groups at each time points were evaluated using the Wilcoxon rank sum test. All tests were two‐sided, with statistical significance set at P < 0.05. All statistical analyses were performed using IBM SPSS Statistics 20.0 (IBM Corp., Armonk, NY, USA).

## 3 Results

### 3.1 Demographics Characteristics

A total of 75 patients with rosacea were finally enrolled, comprising 8 males and 67 females, with a mean age of 32.41 ± 7.51 years and a median disease duration of 2 (0.75, 5) years, and most patients (*n* = 54) had Fitzpatrick skin type 3. Among them, 36 patients were randomized to the control group and 39 to the experimental group. The control group included 4 males and 32 females, with a mean age of 30.6 ± 6.85 years and a median disease duration of 1.5(0.5,3.5) years, while the experimental group consisted of 4 males and 35 females, with a mean age of 34.10 ± 7.80 years and a median disease duration of 4(1,10) years. There were no significant differences in gender and Fitzpatrick skin type distribution between these two groups. However, the mean age of the experimental group was significantly older than that of the control group (*p* = 0.042) (Table 1).

**TABLE 1:** Demographic characteristics.

	Total (*n* = 75)	Control group (*n* = 36)	Experimental group (*n* = 39)	*P*
Sex (female, %)	67(89.33)	32(88.89%)	35(89.74%)	1
Age (years)	32.41 ± 7.51	30.6±6.85	34.10±7.80	0.042
Disease duration (years)	2(0.75, 5)	1.5(0.5,3.5)	4(1,10)	0.04
Fitzpatrick skin type, *n* (%)				0.616
II	12	7	5	
III	54	26	28	
IV	9	3	6	

### 3.2 Efficacy Assessment of Facial Redness, Physiological Parameters and RosQol

#### 3.2.1 The Assessment of Facial Redness

Our study showed that the a* value was significantly higher in the experimental group than that in the control group at baseline [42.77 (38.14, 46.37) vs. 38.67 (31.12, 43.38), *p* = 0.007]. By D28, this difference was no longer significant [36.11 (34.15, 39.85) vs. 38.52 (31.73, 40.45), *p* = 0.567] (Table 2, Figures 2a,b and 3). However, the variation in a* value from baseline to Day 28 (δ a*value) was also significantly greater in the experimental group [4.90 (3.39, 7.72) vs. 1.09 (−0.25, 2.91), *p* < 0.001] (Table 2, Figure 2c). Furthermore, Kruskal–Wallis tests conducted separately for the control and experimental group showed that a* values did not differ significantly across different time points within the control group. In contrast, within the experimental group, the median a* value at D14 was already significantly lower than that at baseline (*p* = 0.016). (Table 2, Figures 2a and 3).

**TABLE 2:** Facial redness, physiological parameters and RosQol in the control and experimental groups during follow‐up.

	Control group	Experiment group	*p* value
a* value (AU)			
Baseline	38.67(31.12,43.38)	42.77(38.14,46.37)	0.007
Day 7	37.16(33.77,41.98)	39.26(35.07,44.68)	0.219
Day 14	37.23(32.64,43.16)	38.90(35.32,42.78)	0.504
Day 28	38.52(31.73,40.45)	36.11(34.15,39.85)	0.567
δ a* value	1.09(‐0.25,2.91)	4.90(3.39,7.72)	<0.001
**Facial red area (AU)**			
Baseline	0.42(0.31,0.54)	0.46(0.38,0.50)	0.618
Day 7	0.43(0.25,0.50)	0.34(0.23,0.45)	0.275
Day 14	0.39(0.31,0.53)	0.26(0.15,0.34)	0.001
Day 28	0.36(0.19,0.50)	0.21(0.13,0.36)	0.040
δ Facial red area	0.09(‐0.02,0.18)	0.22(0.05,0.29)	0.025
Time to a 1‐point reduction in the PSA scale (day)	4(3,8.5)	4(3,6)	0.877
**TEWL (g/h m^2^)**			
Baseline	18.85(13.70,25.55)	22.60(16.60,29.00)	0.062
Day 7	18.75(14.45,25.40)	18.30(13.35,25.20)	0.652
Day 14	15.40(12.70,23.85)	16.90(13.05,23.40)	0.750
Day 28	15.00(12.80,18.40)	15.20(11.35,24.30)	0.975
δ TEWL	2.35(‐1.30,8.15)	6.20(‐0.30,10.30)	0.140
**SCH (AU)**			
Baseline	66.25(57.90,77.10)	62.50(51.90,78.05)	0.637
Day 7	67.75(57.95,78.30)	73.00(59.10,80.20)	0.675
Day 14	71.05(61.20,79.40)	78.30(63.25,88.55)	0.061
Day 28	72.55(61.90,82.30)	82.20(69.30,95.00)	0.020
δ SCH	8.00(3.50,13.00)	17.00(13.00,24.50)	<0.001
**RosQol**			
Day 0	61(56,73.5)	67(55,75.5)	0.454
Day 7	62(52,67)	62(50,68)	0.795
Day 14	54(49.5,67)	51(45,66)	0.265
Day 28	53.5(47.5,64.5)	43(37.5,57.5)	0.002
δ RosQol	5.30(‐3.10,12.10)	16.00(8.80,22.85)	<0.001

*Note*: δ represents the value of the variations from baseline to D28 or from D28 to baseline.

Abbreviations: PSA, Patient's Self‐Assessment; RosQol, Rosacea‐specific Quality‐of‐life instrument; SCH, stratum corneum hydration; TEWL, transepidermal water loss.

**FIGURE 2:**
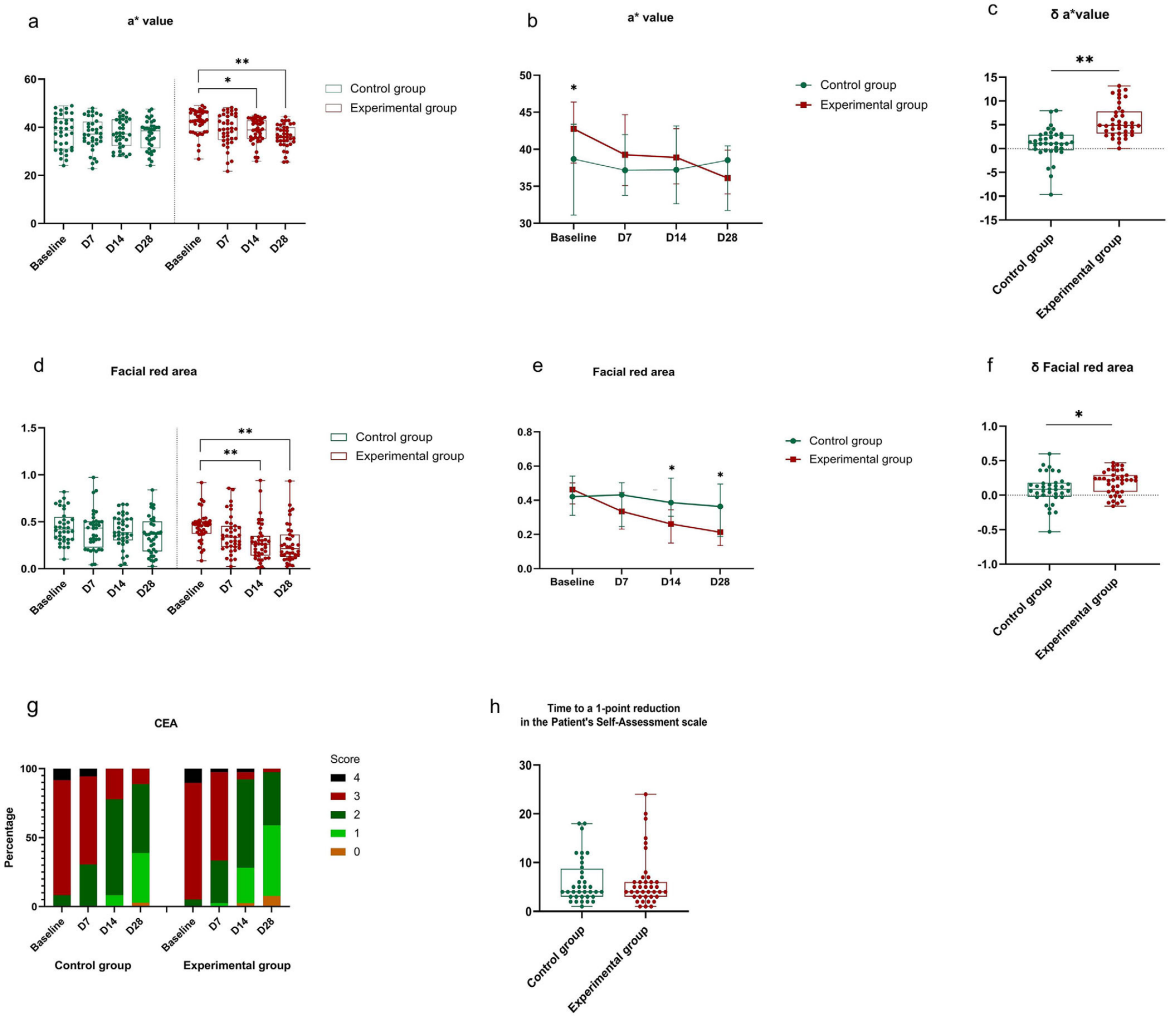
(a–c): The a* value and the variations in a* value from baseline to Day 28 in the control and experimental groups at baseline, D7, D14 and D28; (d–f): The facial red area and the variations in facial red area from baseline to Day 28 in the control and experimental groups at baseline, D7, D14 and D28; (g): The distribution of CEA scores in the control and experimental groups at baseline, D7, D14 and D28; (h): The time to a 1‐point reduction in the PSA scale. Significance is defined as *p* < 0.05: *; *p* < 0.001: ** CEA: Clinician's Erythema Assessment; PSA: Patient's Self‐Assessment.

**FIGURE 3:**
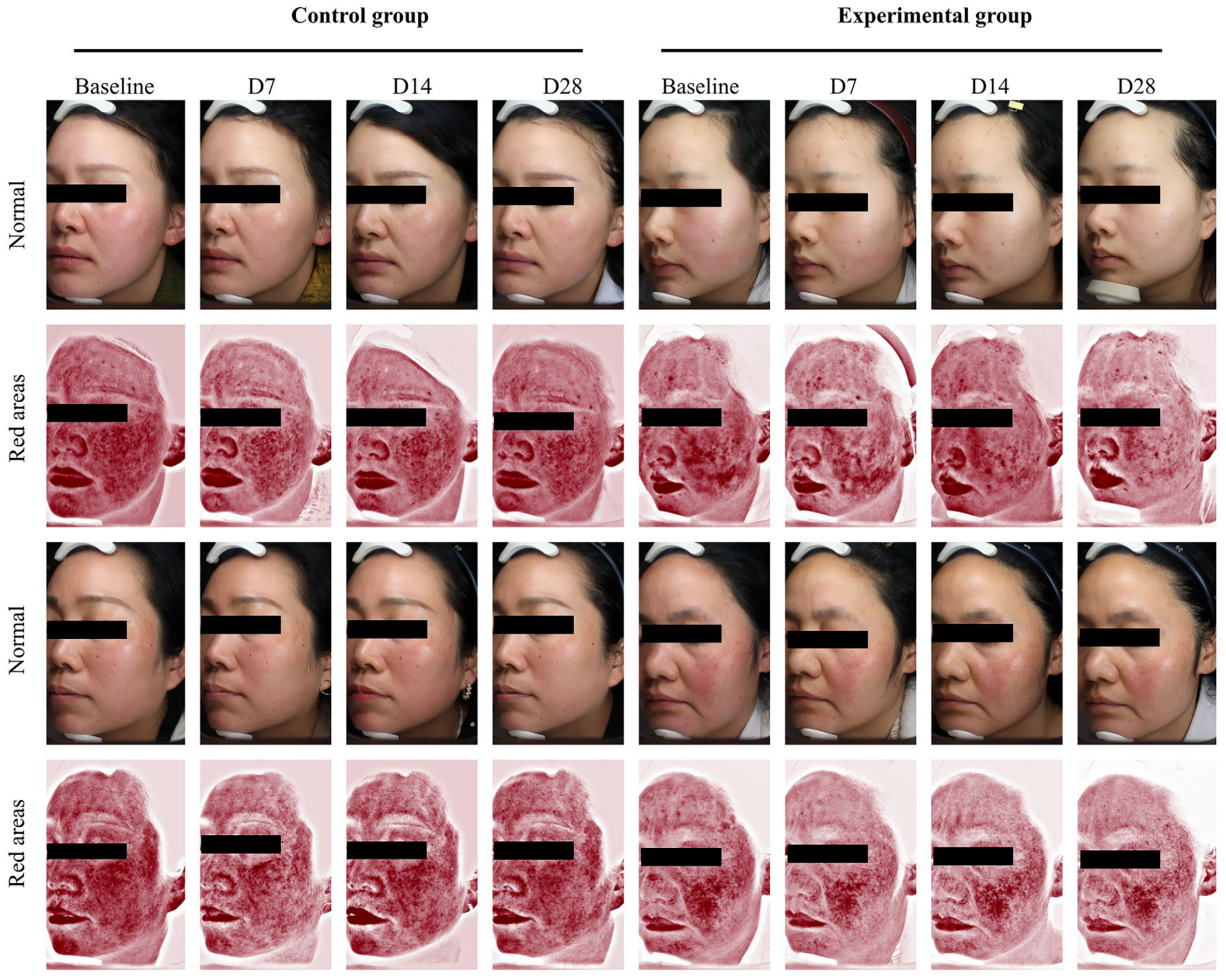
Images of facial red area taken by VISIA.

The facial red area showed no significant difference between these two groups at baseline [0.42 (0.31, 0.54) vs. 0.46 (0.38, 0.50), *p* = 0.618], but was significantly lower in the experimental group than that of control group at both D14 [0.26 (0.15, 0.34) vs. 0.39 (0.31, 0.53), *p* = 0.001] and D28 [0.21 (0.13, 0.36) vs. 0.36 (0.19, 0.50), *p* = 0.040] (Table 2, Figures 2d,e and 3). The variation of facial red area from baseline to D28 (δ facial red area) was also significantly greater in the experimental group compared to the control group [0.22 (0.05, 0.29) vs. 0.09 (−0.02, 0.18), *p* = 0.025] (Table 2, Figure 2f). Similar to the a* value, Kruskal–Wallis tests indicated no significant changes in facial red area across different time points within the control group, while in the experimental group, facial red area was already lower at D14 (*p* < 0.001). (Table 2, Figures 2d and 3).

The Chi‐square test indicated a significant difference in the distribution of CEA scores across different time points in both groups (*p* < 0.001). (Table 3, Figure 2 g) Spearman's rank correlation analysis revealed a significant negative correlation between CEA and time points (*p* < 0.001), with correlation coefficients of *r* = −0.68 (95% CI: −0.77 to −0.58) in the control group and r = −0.77 (95% CI: −0.84 to −0.70) in the experimental group. The Mann–Whitney U test showed that the mean rank of CEA was significantly higher in the control group than that in the experimental group at D28 (42.76 vs. 33.60, *p* = 0.046). However, there was no significant difference between the groups of the time to a 1‐point reduction in the Patient's Self‐Assessment scale [4 (3, 8.5) days vs. 4 (3, 6) days, *p* = 0.877]. (Table 3, Figure 2h).

**TABLE 3:** Changes in the distribution of CEA scores in the control and experimental groups during follow‐up.

	Control group					*P*	Experimental group					*P*
	0	1	2	3	4		0	1	2	3	4	
Baseine	0	0	3	30	3	<0.001	0	0	2	33	4	<0.001
D7	0	0	11	23	2		0	1	12	25	1	
D14	0	3	25	8	0		1	10	25	2	1	
D28	1	13	18	4	0		3	20	15	1	0	

Abbreviation: CEA, Clinician's Erythema Assessment.

#### 3.2.2 The Assessment of Physiological Parameters

No significant difference in TEWL was observed between the control and experimental groups at any time point, including δ TEWL between these two groups (Table 2, Figure 4a–c). Kruskal–Wallis tests showed no significant variation in TEWL over time within the control group. In the experimental group, however, TEWL at D28 was significantly lower than that at baseline [15.20 (11.35, 24.30) vs. 22.60 (16.60, 29.00), *p* < 0.05]. (Table 2, Figure 4a).

**FIGURE 4:**
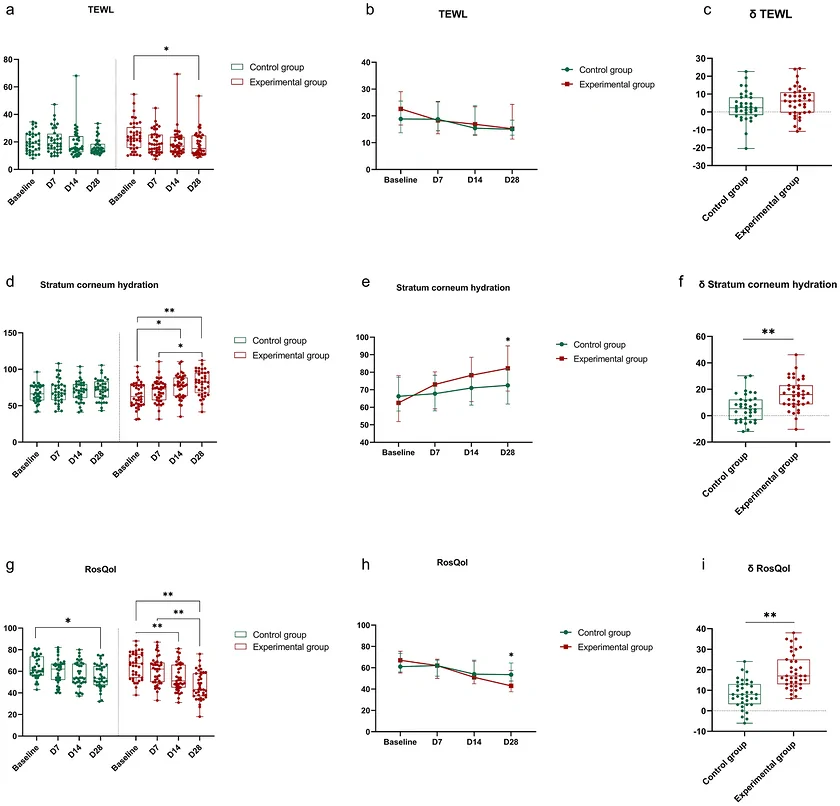
(a–c): The TEWL and the variations in TEWL from baseline to Day 28 in the control and experimental groups at baseline, D7, D14 and D28; (d–f): The stratum corneum hydration and the variations in stratum corneum hydration from Day 28 to baseline in the control and experimental groups at baseline, D7, D14 and D28; (g–i): RosQol scores and the variations in RosQol from baseline to Day 28 in the control and experimental groups at baseline, D7, D14 and D28. Significance is defined as *p* < 0.05: *; *p* < 0.001: **. TEWL: transepidermal water loss; RosQol: Rosacea‐specific Quality‐of‐life instrument.

Stratum corneum hydration (SCH) showed no significant difference between the experimental and control groups at baseline. By D28, however, SCH was significantly higher in the experimental group compared to the control group [82.20 (69.30, 95.00) vs. 72.55 (61.90, 82.30), *p* = 0.02] (Table 2, Figure 4d,e). The increase in SCH from baseline to D28 (δ SCH) was also significantly greater in the experimental group [17.00 (13.00, 24.50) vs. 8.00 (3.50, 13.00), *p* < 0.001] (Table 2, Figure 4f). Kruskal–Wallis tests revealed no significant changes in SCH over time within the control group, while experimental group, SCH at both D14 and D28 was significantly higher than that at baseline. (Table 2, Figure 4d).

#### 3.2.3 The Assessment of RosQol

RosQol scores showed no significant difference between the experimental group and the control group at baseline, D7 or D14. However, at D28, the RosQol score was significantly lower in the experimental group than in the control group [43 (37.5, 57.5) vs. 53.5 (47.5, 64.5), *p* = 0.02], and the variation of RosQol from baseline to D28 (δ RosQol) was significantly greater in the experimental group than in the control group [16.00 (8.80, 22.85) vs. 5.30 (−3.10, 12.10), *P*<0.001] (Table 2, Figure 4g–i). The Kruskal–Wallis test showed that in control group, the RosQol score at D28 was significantly lower than that at baseline, whereas in the experimental group, RosQol scores at both D14 and D28 were significantly lower than at baseline. (Table 2, Figure 4g).

### 3.3 Safety

This study initially enrolled 80 patients, 5 of whom withdrew due to possibly product‐related adverse events (AEs), resulting in a final inclusion of 75 patients. Among these 5 patients, 1 was from the experimental group and 4 were from the control group. The AEs in the experimental group manifested as facial pruritus and erythematous papules, and in the control group, 2 cases presented with facial erythema, stinging, and pruritus; 1 case experienced pruritus and pain, and 1 case showed papules accompanied by stinging and a burning sensation. All AEs resolved spontaneously after discontinuation of the product, and no serious consequences were reported. The incidence of AEs was 2.5% in the experimental group and 10% in the control group, suggesting that the moisturizer used in the experimental group may offer a better safety and tolerability profile compared with the control moisturizer.

## 4 Discussion

This randomized controlled trial found that moisturizer with CAE, panthenol, and ceramide NP could provide significant additional benefits to rosacea patients during minocycline treatment. Compared with the basic moisturizer, moisturizer with CAE, panthenol, and ceramide NP showed favorable tolerability, significantly improved facial erythema and skin barrier function, and markedly enhanced patients’ quality of life.

Moisturizers are mainly composed of occlusives, emollients, and humectants. Occlusives reduce TEWL, increase antimicrobial peptides (AMPs), and promote keratinocyte differentiation. Emollients integrate into the lipid barrier, stimulating the synthesis and secretion of stratum corneum (SC) lipids. Humectants draw moisture from the dermis into the SC and upregulate filaggrin, tight junction proteins, and aquaporin channels [28]. Previous studies have confirmed that appropriate moisturizers benefit inflammatory skin conditions such as atopic dermatitis or acne by improving the skin barrier, reducing inflammation, enhancing tolerance, and increasing adherence to topical treatments without interfering with medicine absorption [21, 29, 30, 31]. A study from Sabine et al. [32] found that combining 0.75% metronidazole gel with a basic moisturizer (Cetaphil Moisturizing Cream) led to significantly greater improvement in erythema, dryness, and skin comfort than 0.75%metronidazole gel alone. Thus, moisturizer use in rosacea not only improves skin tolerance but also enhances treatment efficacy.

In 2005, Rosso et al. [33] proposed that rosacea patients should receive skincare guidance. This was further supported in 2009, when the National Rosacea Society Expert Committee recommended incorporating facial care, including cleansing, moisturizing, and sun protection, into rosacea treatment [1]. Today, proper skincare is widely regarded as an essential component of rosacea management and is explicitly emphasized in recent treatment guidelines or consensus statements from Europe, the United States, and the Asia‐Pacific region [4, 11, 12].

Studies have shown that East Asian skin tends to have a less mature and relatively weaker barrier, resulting in increased skin sensitivity, a greater propensity for irritation, and an enhanced neurosensory response to insults [14, 34]. Moreover, research conducted in China has further indicated that Chinese rosacea patients are more prone to experiencing allergic reactions to skincare products compared to healthy individuals, and they tend to use cleansers, face masks, cosmetics, and beauty salon products more frequently [35, 36, 37]. Therefore, these findings indicate that Asian rosacea patients place greater emphasis on the tolerability of topical agents when selecting treatments [14].

Previous studies have shown that, compared to basic moisturizers, the targeted addition of anti‐inflammatory ingredients, such as niacinamide, licochalcone A, 4‐t‐butylcyclohexanol, or piperonyl glucoside, in skincare products can significantly improve their tolerability in patients with sensitive skin, and notably enhance skin barrier function, alleviate erythema, and improve quality of life in rosacea patients [13, 38]. Furthermore, the anti‐inflammatory effects of key ingredients in the moisturizer used in this study, namely CAE, panthenol and ceramide NP, have also been documented in previous literature. In 2020, Draelos et al. [38] demonstrated that a ceramide‐containing moisturizer significantly improved stratum corneum hydration and increased the levels of ceramides, cholesterol, and free fatty acids, while also alleviating symptoms including pruritus, stinging, burning, facial dryness, roughness, and erythema. In 2025, a study by Gregoriou et al. [39] enrolled 2337 patients who required sedative skin care after irritating treatments and observed that skincare products containing panthenol were highly tolerable and significantly improved patients' quality of life. A separate study also reported that moisturizer containing panthenol could effectively reduce TEWL [40]. Regarding CAE, previous research indicates that this compound, commonly used in wound healing for its anti‐inflammatory and anti‐allergic properties, could block histamine release, inhibit mast cell degranulation, and suppress the release of inflammatory mediators such as tumor necrosis factor (TNF)‐α, interleukin (IL)‐6, myeloperoxidase (MPO), and Cyclooxygenase (COX) ‐2 [41].

In 2025, a study by Su et al. [20] that enrolled 88 patients with sensitive skin demonstrated that moisturizers containing Centella asiatica extract, panthenol, and ceramide NP could significantly improve skin sensitivity, erythema area, and skin barrier function. In line with this observation, our study showed that the same active moisturizer significantly enhanced skin barrier function and reduced both the severity and extent of erythema in rosacea patients. Furthermore, we observed that this combination achieved the additional benefits more rapidly than the basic moisturizer.

Regarding safety, the incidence of AEs with this active moisturizer was 6.82% in a previous study [20]. In our trial, only 1 of 40 patients in the experimental group reported a self‐limiting AE that resolved after moisturizer discontinuation. Moreover, the AE incidence was significantly lower than that in the control group, indicating that this moisturizer may offer superior tolerability in rosacea patients.

This study has several limitations. First, as a single‐center, single‐blind, randomized controlled trial conducted in China, its findings may not be generalizable to other populations without further validation in larger, more diverse cohorts. Second, the absence of patient‐reported outcomes for symptoms like itching and stinging limits the assessment of subjective tolerability. Finally, data based on patient self‐reports may be influenced by individual differences in disease perception and understanding.

## 5 Conclusion

In conclusion, this single‐center, single‐blind, randomized controlled trial demonstrates that the active moisturizer, as an adjunct to standard oral minocycline therapy of rosacea, provides substantial and rapid improvements in patients’ erythema and skin barrier function, which in turn enhances patient quality of life. These findings offer dermatologists an effective new option to improve treatment adherence and clinical outcomes. For patients, this active moisturizer represents a reliable, safe, and gentle daily skincare choice that supports long‐term disease management and quality of life.

## Funding

This work was supported by Galderma

## Ethics Statements

The authors confirm that the ethical policies of the journal, as noted on the journal's author guidelines page, have been adhered to and the appropriate ethical review committee approval has been received. This study was reviewed and approved by Ethics Committee of the Third Affiliated Hospital of Chongqing Medical University (IRB number: 2024/79), and registered in the Chinese Clinical Trial Registry (ChiCTR2500096690).

## Consent

The authors attest to obtaining written patient consent for the publication of recognizable patient photographs or other identifiable material, with the understanding that this information may be publicly available.

## Conflicts of Interest

The authors declare no conflicts of interest.

## Data Availability

The data that support the findings of this study are available on request from the corresponding author. The data are not publicly available due to privacy or ethical restrictions.
